# Impaired circulating myeloid CD1c+ dendritic cell function in human glioblastoma is restored by p38 inhibition – implications for the next generation of DC vaccines

**DOI:** 10.1080/2162402X.2019.1593803

**Published:** 2019-04-13

**Authors:** Jason Adhikaree, Hester Ann Franks, Constantinos Televantos, Poonam Vaghela, Aanchal Preet Kaur, David Walker, Marc Schmitz, Andrew Mark Jackson, Poulam Manubhai Patel

**Affiliations:** a Division of Cancer and Stem Cells, Host-Tumour Interactions Group, UK; b Children’s Brain Tumour Research Centre, University of Nottingham, Nottingham, UK; c Institute of Immunology, Medical Faculty Carl Gustav Carus, TU Dresden, Dresden, Germany; d National Center for Tumor Diseases, University Hospital Carl Gustav Carus, TU Dresden, Dresden, Germany; e German Cancer Consortium (DKTK), Dresden, Germany; f German Cancer Research Center (DKFZ), Heidelberg, Germany

**Keywords:** Glioblastoma, dendritic cell, signalling, immunotherapy, mitogen activated protein kinase

## Abstract

Current treatments for glioblastoma (GBM) have limited efficacy and significant morbidity and therefore new strategies are urgently needed. Dendritic cells have the power to create anti-tumor immune responses. The greater potency of circulating dendritic cells (DC) over laboratory-generated monocyte-derived DC makes them exciting new immunotherapeutic candidates. To determine the immune status of GBM patients we initially investigated the frequency and function of circulating DC subsets. Furthermore, we tested the therapeutic potential of inhibiting the p38 mitogen-activated protein kinase pathway (p38i) in circulating DC to overcome DC dysfunction.

GBM patients (n = 16) had significantly reduced numbers of the major myeloid circulating dendritic cell (cDC2) and plasmacytoid DC vs healthy controls; 1736 vs 4975 (p = 0.028) and 893 vs 2287 cells/mL (P = <0.001) respectively. This inversely correlated with dexamethasone (Dex) dose in a log-linear model, and disease status. Patients’ cDC2 were immature with impaired interleukin (IL)-12 secretion, reduced IL-12:IL-10 ratio, and low HLA-DR and CD86 expression. Exposure of healthy donor cDC2 to Dex or GBM cell lysate resulted in a similar low IL-12:IL-10 ratio. Inhibition of p38 restored the IL-12:IL-10 balance in Dex or tumor lysate-conditioned healthy cDC2 and enhanced T-cell proliferation and interferon-gamma (IFNγ) production. Importantly, patient-derived cDC2 showed a similar reversal of DC dysfunction with p38i. This study demonstrates the therapeutic potential of developing the next generation of DC vaccines using enhanced p38i-conditioned cDC2. We will therefore shortly embark on a clinical trial of adoptively transferred, p38 MAPK-inhibited cDC2 in adults with GBM.

## Introduction

Glioblastoma Multiforme (GBM), the most common primary malignant brain tumor in adults, is a devastating disease that inevitably becomes resistant to surgery, chemotherapy, and radiation. The extraordinary power of enhancing the immune system to combat the chemo-radiotherapy resistant tumor, melanoma, affirms the potential for re-directing immune responses against GBM. Furthermore, such approaches have the added advantage of combining initial anti-cancer cytotoxic immunity with durable memory responses.^1,2^

Dendritic cells (DC) play a central role in the anti-cancer immune cycle^3^ orchestrating adaptive immune responses by antigen uptake, presentation, and co-stimulation of effector T cells. They are, therefore, critical for the detection of cancers as “dangerous” and initiating antigen-specific T-cell responses.^4^ Additionally, DC control the cytotoxic activity of, and interferon-gamma (IFNγ) production by, natural killer (NK) cells, which also serve important roles against cancer.^5^ However, tumors have evolved numerous mechanisms to suppress DC function and evade immune recognition.^6,7^

In GBM patients, the hostile tumor microenvironment comprises, amongst other factors, interleukin (IL)-10, transforming growth factor-beta (TGF-β), and indoleamine 2,3-dioxygenase (IDO), which potently suppress the function of DC.^6,8^ Furthermore, this patient group is often treated with corticosteroids, mainly dexamethasone (Dex), to control cerebral edema. Such corticosteroids markedly impact on DC differentiation, maturation, cytokine secretion and induction of lymphocyte responses.^9,10^ These effects are seen with laboratory-generated, monocyte-derived DC (moDC) and have also shown to decrease circulating myeloid and plasmacytoid DC (pDC) numbers in non-cancer patients.^10^

Current strategies to overcome this DC immune dysfunction in GBM have focussed on the adoptive transfer of *ex-vivo* generated autologous moDC.^11-13^ However, clinical results in several tumor types, including glioma, have been disappointing, with benefit only seen in selected patients, and objective response rates rarely exceed 15%.^14^ Therefore, recent interest has turned to using primary circulating DC isolated directly from peripheral blood. These have been shown in experimental systems to be superior to moDC in activating the adaptive immune response.

There is a paucity of data concerning circulating DC in GBM. The distinct subsets of circulating primary DC have different phenotypes with non-overlapping functions. In humans, DC are categorized as classical DC (cDC) and pDC. Classical DC are myeloid in origin, can be further subdivided by their expression of CD1c+ (BDCA-1) and CD141+ (BDCA-3) and were recently renamed cDC2 and cDC1 respectively.^15-17^ SlanDC are a non-classical subset of human myeloid DC that share some characteristics with monocytes, notably their proinflammatory properties and association with inflammatory diseases.^15,18^ Recently, the study of Villani and colleagues employed single-cell RNA sequencing to refine the definition of natural DC suggesting that human DC are even more heterogeneous than previously appreciated.^19^ In man, cDC2 cells are particularly important as they are the most abundant classical myeloid DC, produce the Th1-polarising cytokine IL-12, efficiently cross-present antigens and activate T-helper 1 (Th1) and CD8+ cytotoxic T lymphocyte (CTL) responses.^20^ In contrast to cDC1 cells, that exhibit potent ability to cross-present antigen, cDC2 are over 10-times more frequent in the periphery and this makes them (and pDC) viable candidates for adoptive immunotherapy. Importantly, recent advances in clinical-grade cell isolation technology mean that cDC2 and pDC (but not cDC1) can be harvested from leukapheresis using magnetic cell sorting. This therefore provides an opportunity for the next generation of DC-based adoptive immunotherapy with natural DC.^21^ Indeed, in addition to functional superiority over moDC, circulating DC have the distinct advantage that they do not require lengthy laboratory differentiation, but rather can immediately be loaded with antigen and matured. Results from a recent phase I/II trial of adoptively transferred cDC2 in advanced melanoma patients produced *de novo*, antigen-specific T cell responses, and objective tumor regression.^22^

A previous study by Gousias and colleagues showed decreased numbers of circulating DC in glioma patients.^23^ However, the phenotypic characteristics of the DC subsets, and their function was outside the scope of that study. There is an urgent need to understand the function of circulating DC function in GBM patients, in particular, mechanisms to alleviate tumor mediated DC dysfunction. Obtaining this information is important for future considerations of the adoptive transfer of cDC2 in adult GBM. Therefore, we determined the abundance of circulating DC and their associated phenotypic and functional characteristics in adult GBM patients.

The function of DC is differentially regulated by complex networks of intracellular signaling pathways.^24-27^ Intriguingly, these pathways do not always serve the same function in all subsets of DC. This is well illustrated by the p38 mitogen-activated protein kinase (MAPK) pathway that differentially controls expression of the Th1-polarising cytokine IL-12 from moDC and cDC2.^25^ Inhibiting p38 MAPK (p38i) in cDC2, but not moDC, results in high levels of IL-12 in the absence of IL-10.^25^ This phenotype is ideally suited to promote the Th1 responses required for optimal priming of cytotoxic T lymphocytes (CTL) and the activity of NK cells.^28,29^

Here we show a novel detailed analysis of circulating DC subsets in patients with GBM. Furthermore, we demonstrate for the first time that tumor mediated DC dysfunction can be reversed in heavily treated patients by p38i of myeloid DC. Importantly, in this patient group, dexamethasone mediated dysfunction is also reversed using p38i. This discovery paves the way for the next generation adoptive therapy using p38i-treated autologous circulating DC to treat GBM patients.

## Results

### Patient characteristics

To explore if there were sufficient circulating DC numbers for autologous vaccine treatment we recruited patients at various stages of treatment (Table 1). The cohort was typical for GBM patients undergoing active treatment. Sixty-nine percent were taking steroids, while all but two patients were undergoing treatment with either chemotherapy and/or radiotherapy. Sixty-three percent had the residual disease and 56% had a gross neurological deficit. The mean age of GBM patients was 59 yr, compared to 49 yr in the healthy controls. The median survival in the cohort was 14.0 months and had a median performance status of one.

**Table 1.** Patient characteristics who were recruited for circulating DC analysis.

Glioblastoma Patients n = 16	
*Age (years)*	
Median (Range)	59 (37–74)
*Sex*	
Male	13
Female	3
*ECOG Performance Status*	
0	1
1	13
2	2
*Treatment at DC analysis*	
Chemoradiation	5
Radiotherapy	2
Adjuvant Temozolomide	3
PCV chemotherapy	4
Remission	2
*Type*	
Primary	14
Secondary	2
*Dexamethasone dose*	
None	5
0.5-2 mg	4
>2 mg-4 mg	6
>4 mg	1
*Residual Disease*	
Yes	10
No	6
*Symptomatic Neurology*	
Yes	9
No	7
*Survival (months)*	
Median (range)	14.0 (3.8–138.5)
*IDH1 status*	
Unknown	5
Mutated	1
Wild type	10

ECOG = Eastern co-operative oncology group. PCV = procarbazine, lomustine and vincristine chemotherapy. IDH1 = isocitrate dehydrogenase 1

### Alterations in circulating DC numbers in patients with GBM

**Figure 1.**
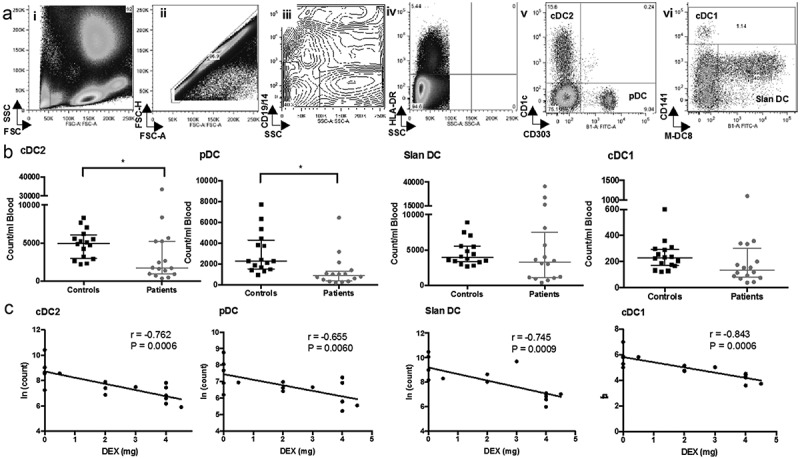
Enumeration of circulating DC in GBM patients and healthy controls. (a) Flow cytometry was used to enumerate circulating DC. Briefly, whole blood was stained and red blood cells lysed prior to acquisition on a flow cytometer. (i) Shows the typical forward (FSC) and side scatter (SSC) of the leukocyte populations. Doublet events were excluded by plotting FSC height against area (ii). The exclusion of B cells, monocytes and granulocytes were achieved by gating CD14-/19- and side scatter low events (iii). HLA-DR high events, a hallmark of DC, were then selected (iv). Lineage markers for cDC2 (CD1c+), pDC (CD303+) (v), cDC1(CD141^high^), and slanDC (Slan+) (vi) were then identified against isotype controls (purple gates). (b) Absolute numbers of circulating DC in GBM patients compared with healthy controls.(c) Correlation of steroid dose on the day of blood acquisition to DC counts (in natural log for parametric analysis). Pearson's correlation coefficient (r) and significance are shown. Dex dose is associated with lower numbers of all subsets of circulating DC * = p < 0.05.

We determined the abundance of the major circulating DC subsets using flow cytometry on whole blood in GBM patients (n = 16) and healthy adults (n = 16) (Figure 1). As expected, cDC2 and slanDC cells were the most frequent circulating DC subsets identified. The cDC2 and pDC subsets were significantly reduced in patients (cDC2 median 1736 vs 4975 cells/mL p = 0.028, pDC 893 vs 2287 cells/mL p < 0.001, patients vs controls, respectively). In contrast, there were equivalent numbers of the slanDC in patients and controls (3296 vs 3936 cells/mL, respectively; p = 0.379). The myeloid cDC1 subset had a non-significant trend towards lower numbers (135 vs 228 cells/mL, patients vs controls p = 0.078). Even in the context of this sample size, it was possible to identify a proportion of patients with similar numbers of circulating DC as healthy controls (Figure 1(b)).

We undertook a preliminary univariate analysis and found that patients with higher circulating DC numbers had increased survival (cDC2 p = 0.019; pDC p = 0.004; and cDC1 p = 0.009). Higher numbers of cDC2 were associated with no steroid use at the time of blood acquisition (p = 0.005), no residual disease (p = 0.028), a secondary GBM (p = 0.002) and in remission (p = 0.01).

### Association between reduced number of DC and dexamethasone

An association existed between the number of circulating DC subsets and the Dex dose at the time of blood acquisition. In all DC subsets, there was a significant linear relationship between increasing dose of Dex and reduced number of DC; cDC2 p = 0.0006; pDC p = 0.0060; slanDC p = 0.0009 and cDC2 DC p = 0.0009 (Figure 1(c)). In the cDC2 subset, the Pearson coefficient was −0.762, indicating a strong correlation between cell number and dose of Dex. This remained significant after adjusting for confounders of primary or secondary GBM and current treatment. Age, sex, residual disease, overt neurology and performance status were not significant confounders in this analysis.

### Altered phenotype of circulating DC

**Figure 2.**
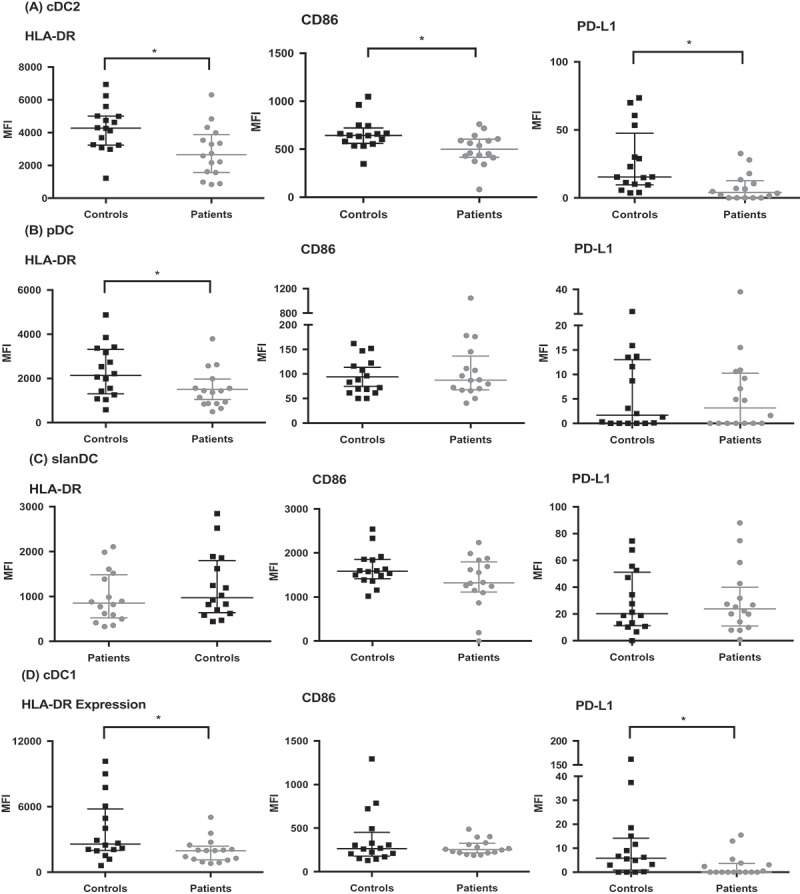
Circulating DC have altered phenotype in GBM patients compared to healthy controls. DC were analyzed by flow cytometry as in Figure 1. Subsequently, the expression of HLA-DR, CD86, and PD-L1 was determined. The change in median fluorescence intensity (MFI) was calculated by subtracting the MFI of the phenotype marker from its isotype control. (a) shows lower HLA-DR, CD86 and PD-L1 expression in cDC2 of patients compared to controls. There was also lower HLA-DR expression in pDC (b) and cDC1 (d). *p < 0.05.

The expression of HLA-DR on patients’ circulating cDC2, cDC1, and pDC cells was significantly lower than with healthy controls (p = 0.009, 0.029, and 0.047, respectively; Figure 2). Additionally, CD86 expression was significantly lower on patients’ cDC2 cells (p = 0.01). Expression of PD-L1, was significantly lower in GBM patients in both the cDC2 (p = 0.003) and cDC1 (p = 0.015) subsets. Overall, low HLA-DR and/or CD86 represent a less mature phenotype of the DC. Furthermore, immature DC stimulate T cell tolerance, and hence this is a potential mechanism for tumor tolerance in GBM.^4,6^ In contrast to the other subsets studied, the phenotype of slanDC was unaffected in GBM patients.

### Functional impairment of cDC2 cells isolated from GBM patients

**Figure 3.**
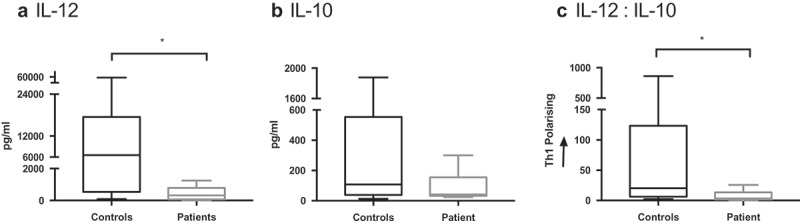
cDC2 cytokine release in GBM patients is suppressed compared to healthy controls. cDC2 were isolated from whole blood of healthy controls (n = 9) and GBM patients (n = 6), then matured *ex-vivo* with polyI:C and R848. Cytokine release was determined after 24 hrs by ELISA. IL-12 secretion was significantly suppressed (a) and IL-10 has a trend to lower levels (b) in patients compared to healthy controls. Overall the ratio of IL-12 to IL-10 would skew away from Th1 polarisation (c). *p < 0.05.

Clinical grade isolation of the cDC2 subset is now possible, and the utility of these cells as an autologous vaccine is under clinical investigation in prostate cancer and malignant melanoma.^22,30^ The capacity to produce IL-12 to support Th1-dependent CTL responses is an important feature of cDC2 cells.^28,31^ When cDC2 were isolated from patients (n = 6) and healthy controls they secreted heterogeneous amounts of IL-10 and IL-12 (Figure 3). IL-12 secretion from cDC2 was significantly impaired in GBM patients (median 6541 vs 322 pg/mL, respectively; p = 0.0245). However, IL-10 secretion was equivalent, with a median of 108 pg/mL in healthy volunteers compared to 41 pg/mL (ns). Overall, the ratio of IL-12: IL-10, which determines CD4 + T cell polarisation, significantly favored Th1 response in healthy controls compared to GBM patients (p = 0.032).^32^ This represents an impairment in GBM patients to orchestrate appropriate anti-tumor immune responses.

### Exposure of healthy DC to tumor lysate (TL) or Dex replicates DC dysfunction observed in GBM

**Figure 4.**
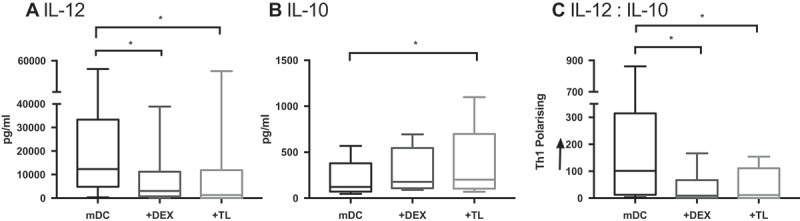
cDC2 cytokine release from healthy volunteers is skewed when exposed to Dex and TL. cDC2 were isolated from healthy volunteers (n = 9). DC were exposed *ex-vivo* to Dex or TL prior to maturation with polyI:C and R848. Cytokines were measured 24 hrs later by ELISA. Dex and TL exposed DC had significantly suppressed IL-12 secretion compared to mature DC (mDC) (a). TL exposure also significantly increased the immunosuppressive cytokine IL-10 secretion (b). Overall the ratio of IL-12:IL-10 secretion by Dex- and TL-cultured DC was significantly skewed away from Th1 polarisation, in comparison to mDC (c). *p < 0.05.

To investigate factors contributing to DC dysfunction in GBM, we tested the hypothesis that Dex or GBM tumor lysate (TL) altered the IL-12/IL-10 cytokine balance of cDC2. The secretion of IL-12 from healthy cDC2 (12309 pg/mL) was significantly suppressed following exposure to either Dex (3050 pg/mL p = 0.0094) or TL (1294 pg/mL p = 0.0008; Figure 4). Furthermore, whilst Dex had no significant impact on IL-10 (p = 0.1187), exposure to TL increased its secretion from 121.4 pg/mL to 200.3 pg/mL (p = 0.0044). Overall these factors significantly skewed the cytokine release away from a classical Th1-polarising profile (Dex p = 0.0094 and TL p = 0.0008).

### Inhibition of p38 MAPK reverses Dex and TL induced dysfunction in cDC2

**Figure 5.**
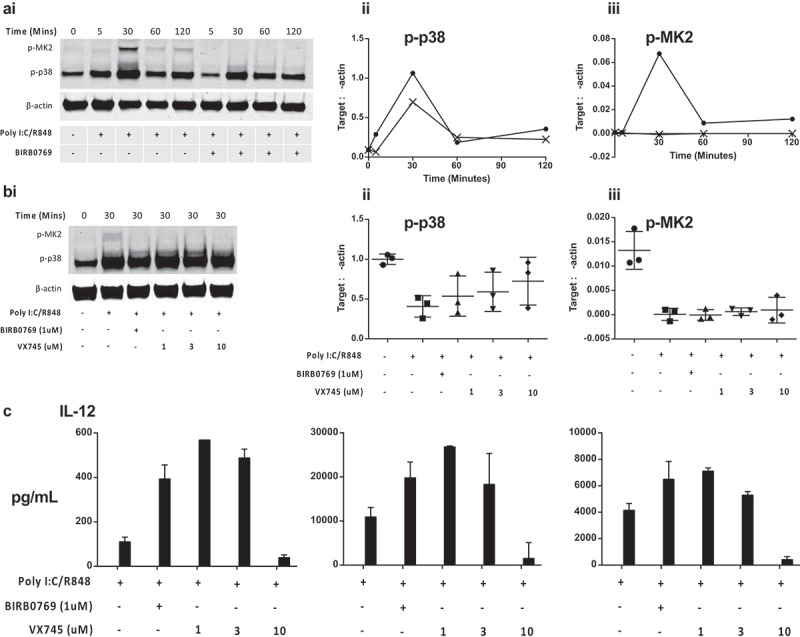
Analysis of the p38 MAPK-MK2 signaling pathway in cDC2 cells. (a): p38i blocks downstream MK2 phosphorylation: (i) Western blot shows the time course of both p38 and MK2 phsophorylation in cDC2 cells and identifies peak activation at 30 min. Isolated cDC2 were activated as indicated (polyI:C/R848) in the presence of absence of BIRB0796 for the indicated periods. Relative expression to actin was obtained from Western blots to show (ii) phospho-p38 MAPK and (iii) phospho-MK2 levels in activated cDC2 in the absence (circle) or presence (cross) of BIRB0796. (b): Comparison of two p38 MAPK inhibitors: (i) Western blot showing the level of phospho-p38 MAPK or MK2 30 min after activation of cDC2 cells. Cells were activated in the absence or presence of BIRB0796 (1 µM), or VX745 at a range of concentrations. Relative quantification of 3 independent-donor Western blot experiments showing inhibition of (ii) phospho-p38 MAPK and (iii) phospho-MK2 levels in activated cDC2. Error bars indicate mean and 1 standard deviation. (c): Inhibition of p38 MAPK enhances IL-12 responses: Isolated cDC2 were treated with the indicated concentrations of BIRB0796 or VX745 prior to activation with polyI:C/R848 and IL-12 secretion determined by ELISA after 24 hrs.

Previously we established that the p38 MAPK pathway serves a special function in cDC2 cells where p38i increases IL-12 expression, whereas p38i inhibits IL-12 in moDC and slanDC.^25,33^ We therefore hypothesized that inhibition of p38 MAPK in cDC2 would reverse the immunosuppressive effects of Dex and TL and initially characterized the impact of selective p38 MAPK inhibitors on the p38-MK2 pathway. Activation with Poly I:C/R848 increased phosphorylation of p38 MAPK and its downstream target, MK2, by 30 min (Figure 5(a)). As expected, BIRB0796 (1 µM) did not markedly inhibit p38 phosphorylation but inhibited p38 function as shown by the ablation of MK2 phosphorylation (Figure 5(a)). To confirm that inhibition of MK2 phosphorylation was a class-effect for selective p38 inhibitors and not restricted to BIRB0796 we determined that a second selective p38 MAPK inhibitor, VX745, also prevented p38 MAPK function. When isolated cDC2 were treated with 1-10 µM VX745 MK2 phosphorylation was prevented (Figure 5(b)). Relative quantification of Western blots from three independent donors confirmed the negative impact of BIRB0796 or VX745 on the p38-MK2 pathway (Figure 5(bii and iii). Treatment of cDC2 cells with 1 µM of either p38 MAPK inhibitor enhanced IL-12 secretion (p < 0.05 comparing PolyI:C/R848 alone to 1 µM p38 MAPK inhibitor, Figure 5(c)). Concentrations of VX745 >1 µM did not further enhance cytokine levels and at 10 µM ablated the response.

**Figure 6.**
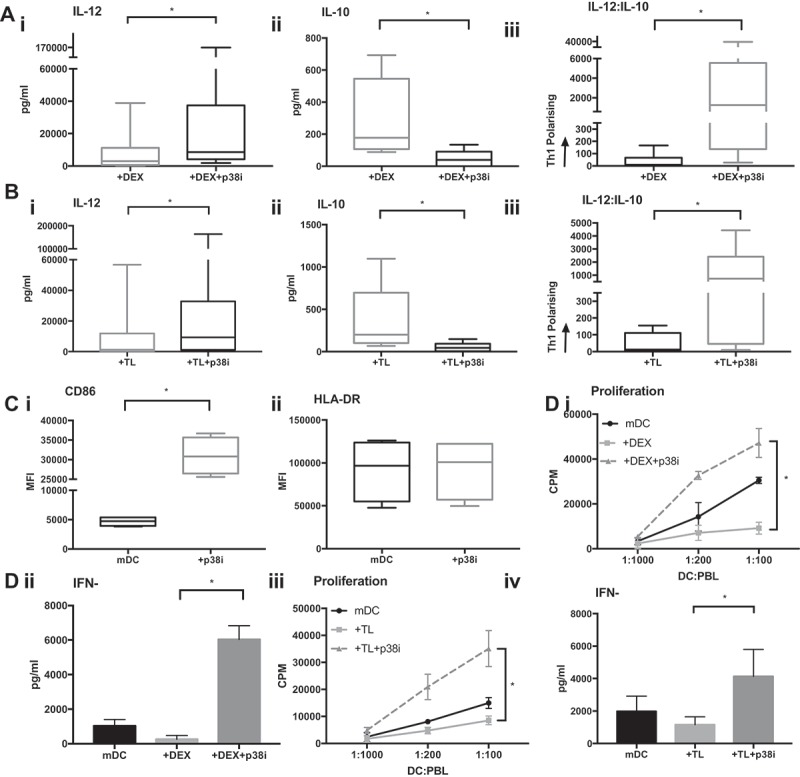
p38i restores the IL-12:IL-10 balance from the Dex or TL treated cDC2. (a): Reversal of the suppressive effects of Dex. cDC2 isolated from healthy volunteers exposed to Dex with/or without p38i (n = 9) showed significantly higher IL-12 (i), reduced IL-10 (ii) and a favorable skewing towards Th1 polarisation with p38i (iii). Figure 6 (b): Reversal of adverse effects of TL on cytokine. Healthy donor cDC2 (n = 9) exposed to TL showed higher IL-12 (i), reduced IL-10 (ii) and a favorable skewing towards Th1 polarisation with p38i (iii). (c): Improved phenotype of DC with p38i treatment. Isolated cDC2 were matured with R848 and polyI:C with/or without pre-incubation with p38i (n = 4). CD86 expression was significantly increased in p38i cDC2 compared to matured cDC2 (i) while HLA-DR expression was unchanged. (d): Enhanced T-cell stimulatory capacity of DC following p38i. Isolated cDC2 were exposed to Dex and TL with/without p38i. DC were then co-cultured with lymphocytes for 5 days (n = 3). p38i restored and enhanced cDC2 stimulatory capacity in MLR above normal levels (mDC). Data from a representative donor is shown (i and iii). A significant increase in IFNγ secretion suggested a Th1 polarisation results as shown in the representative example (ii and iv).

Inhibition of p38 MAPK significantly increased IL-12 expression in both Dex (from median 3050 to 8555 pg/mL; p = 0.0011) and TL treated DC (from median 1294 to 9314 pg/mL; p = 0.0175). Furthermore, p38i reduced secretion of the regulatory cytokine IL-10 (Dex median 176.9 to 39 pg/mL; P = 0.0019, TL median (200.3 to 45.3 pg/mL; p = 0.0006). As such, inhibition of p38 MAPK skewed the cytokine release profile of cDC2 towards a pattern associated with Th1 responses (Figure 6(a,b)).

The functional activity of p38i cDC2 was also determined using MLR. When healthy donor cDC2 were exposed to Dex or TL, T-cell proliferation was significantly suppressed (p = 0.0085 and p = 0.0495, respectively). However, inhibition of p38 MAPK restored the capacity of Dex or TL-treated DC to stimulate T-cell expansion (p < 0.0001 and p < 0.0001, respectively) (Figure 6(d)). Exposure of cDC2 to Dex or TL suppressed IFNγ production in the MLR. However, inhibiting p38 MAPK in cDC2 cells recovered the secretion of IFNγ in the MLR to levels markedly greater than seen with control cells. In contrast, no changes in IL-10 production were observed (not shown). We noted that whilst HLA-DR levels were unaffected by p38i (Figure 6(c)), there was a six-fold increase in CD86 expression (p = 0.001).

### P38 MAPK inhibition of GBM patients cDC2 recovers cytokine function

Having established a model of cDC2 dysfunction in GBM and recovery by p38i in healthy donor cells, we investigated the consequence of inhibiting p38 MAPK in GBM patient-derived cDC2 cells. A further six patients were recruited undergoing first-line treatment with either radiotherapy (one patient), chemoradiotherapy (four patients) or adjuvant Temozolomide (n = 1), had exposure to Dex (n = 4) and residual disease (n = 5) and cDC2 cells were isolated with >95% purity.

**Figure 7.**
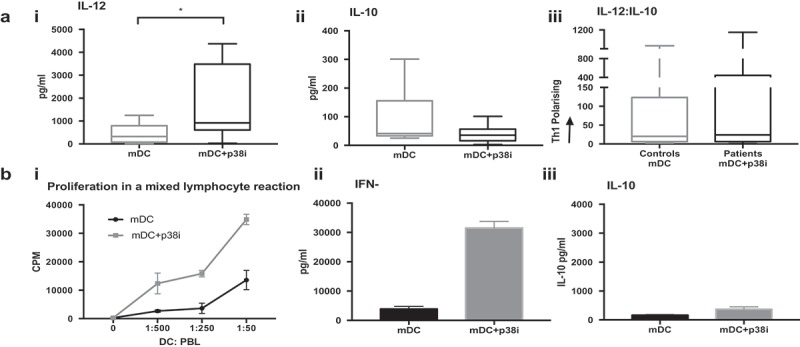
Patients cDC2 function is improved by inhibiting p38 MAPK. (a): p38i skews patients’ cDC2 to favorable cytokine release profile: Treatment of GBM patients’ (n = 6) cDC2 with p38i (mDC+p38i) (i) improved IL-12 secretion, (iii) and restored the IL-12: IL10 balance without affecting IL-10 (ii), compared to maturation alone (mDC). (b): Patient p38i cDC2 had the superior proliferative capacity with IFNγ signature: cDC2 isolated from GBM patients were treated ex-vivo with p38i and matured for 24 hrs. DC were then co-cultured with allogeneic peripheral blood lymphocytes in a mixed lymphocyte reaction for 5 days. Proliferation data from a representative donor is shown. DC stimulatory capacity was superior with p38i treatment (i) and the resulting supernatant was rich in IFNγ (ii) and low in IL-10 (iii). *p < 0.05.

The median number of isolated cDC2 obtained was 3625 cells/mL (range 1,125–7,160 cells/mL), which comprised 0.52% of the peripheral blood mononuclear cells (PBMC), range 0.35–1.50%. Inhibition of the p38 MAPK pathway resulted in a favorable alteration in the balance of cytokines produced by activated cells (Figure 7). Interleukin-12 secretion significantly increased from a median of 321.9 to 920.6 pg/mL (P = 0.0312) upon treatment with p38i. The secretion of IL-10 was low (41.1 pg/mL) and unaffected by p38i (35.6 pg/mL, ns). Importantly, after p38i, the ratio of IL-12:IL-10 was skewed in favor of Th1 responses and was similar to that found in isolated healthy donor cDC2 (Figure 7(a)). Furthermore, the T-cell stimulatory capacity of p38i cDC2 from patients was enhanced (Figure 7(bi), with increased proliferation and IFNγ release from the MLR driven by matured p38i-cDC2, compared to that stimulated by matured cDC2 only (Figure 7(bii). IL-10 secretion remained unaffected (Figure 7(biii).

## Discussion

There is an urgent need for new therapies to treat patients with advanced GBM. This is the first detailed analysis focusing on the abundance, phenotype, and function of circulating DC in GBM. Furthermore, we go on to demonstrate a mechanism for rescuing dysfunction with clear therapeutic potential for the next generation of dendritic cell immunotherapy.

We found that there were fewer circulating cDC2 and pDC cells, and a trend towards fewer cDC1 in GBM patients, whilst the slanDC subset were unaffected. This is in keeping with less detailed data on circulating DC numbers in GBM patients.^23,34^ A cohort of cancer patients, including six with GBM, were found to have suppressed numbers of CD11c+ and CD123+ DC subsets.^34^ Since that study, CD11c+ DC have been further divided into the cDC2 (CD1c+) and cDC1 (CD141+) subsets analyzed in our study. This is relevant since we now appreciate the specialized function of individual DC subsets.^15-17,19^ Furthermore, we employed a methodology with minimal manipulation in which cells were stained in whole blood and avoided errors introduced by PBMC isolation in the aforementioned study. Importantly we only required 4 ml of blood for our study, therefore this allowed more accurate measurement of both the relative frequency and absolute number of DC in blood. Another group also looked at the cDC2, cDC1 and pDC subsets in a cohort of gliomas which included 17 GBM patients.^23^ Our findings are supportive of theirs, with suppressed cDC2 and pDC number. However, we were able to perform phenotypical evaluation including CD86, HLA-DR, and PD-L1. We found the phenotype of circulating DC subsets was altered in GBM patients with significant reductions in HLA-DR and CD86 expression. A low HLA-DR and/or CD86 expression represents an immature DC phenotype and would reasonably be expected to result in T cell tolerance.^6,7^ Furthermore, our novel data on CD86 and PD-L1 expression is important, not just for determining their maturation status, but in the context of immunotherapy currently targeting co-stimulatory/regulatory ligand pairs.

The use of Dex in GBM is independently associated with reduced effectiveness of treatment and overall survival.^9,35^ Indeed, in other settings the immune system plays a vital role in the success of chemotherapy and radiotherapy which induces immunogenic cell death.^36^ Dex is immunosuppressive and studies in the transplantation field showed the exposure of human cDC2 to Dex induces tolerogenic DC.^37^ The present study represents one of the first functional studies of the cDC2 subset in GBM and identifies suppressed IL-12 responses in these patients. The decreased IL-12:IL-10 ratio in activated cDC2 would be predicted to result in weaker Th1 and NK responses. Whilst this study does not attempt to define the driving force behind these changes, we successfully replicated this phenotype by exposing healthy cDC2 to Dex or TL.

We established a favorable effect of p38i in cDC2 for IL-12 production, increased CD86 expression and capacity to promote T-cell proliferation with high levels of IFNγ. However, it remains to be determined what the impact of p38i is on other important aspects of cDC2 function including antigen-uptake, antigen-processing, and cross-presentation to T-cells, and migratory capacity. Further studies are currently underway to address these important features of DC biology. A more detailed understanding of the impact of inhibiting specific signaling pathways in natural DC should include detailed transcriptional analysis (RNASeq) and important information could be obtained from patients receiving oral p38i for other inflammatory conditions using single-cell RNASeq on whole blood or isolated cDC2.

The canonical understanding of the p38 MAPK pathway is that it promotes expression of inflammatory genes.^38^ BIRB0796 is a potent inhibitor of all 4 p38i isoforms with 330-fold greater selectivity versus c-Jun N-terminal kinase 2, weak inhibition for c-RAF, and insignificant activity against extracellular regulated kinase.^39^ It also has a particularly slow dissociation rate constant and this is thought to contribute to its potency. In cDC2, the p38 pathway represses IL-12 expression and consequently inhibition of p38 increases IL-12 secretion.^25^ We made similar findings with additional selective p38 MAPK antagonists, VX-745 and SB203580 (not shown) showing that our findings are consistent with a p38 MAPK inhibitor “class-effect” and not unique to one compound. Newer compounds with improved safety profiles have now been developed (e.g. PH-797804) and these may be suitable candidates for phase I studies of adoptively transferred, p38i cDC2 cells in GBM.^40^ In consideration of a clinical trial of p38 MAPK inhibited, adoptively transferred cDC2 it is likely that alternate MAPK inhibitors would be employed that are highly specific for p38 MAPK and are irreversible/have slow off-rate. It is worth mention that preliminary experiments (n = 2) show that when cDC2 were briefly (1 hr) exposed to BIRB0796 (1 uM), washed and activated, their profile of IL-12/10 expression conformed to that obtained with chronic exposure to MAPK inhibitor (data not shown). Importantly, in contrast to most clinical p38i that are typically formulated for oral or inhaled delivery, our proposed clinical study will require a GMP compound already suitably formulated (i.e. i.v.) for delivery to isolated cDC2 cells in a cell-processing facility.

Obtaining sufficient cell numbers remains a persistent challenge in the development of adoptive immunotherapy with circulating DC. Given that leukopheresis typically yields 7 × 10^9^ PBMC, and in our hands, isolated cDC2 comprised 0.52% of PBMC, we could expect 3.6 × 10^7^ cDC2 per leukopheresis.^41^ A recent melanoma phase I trial with cDC2 cells used 3–10 × 10^6^ cells per treatment.^22^ Hence, even though cDC2 numbers are decreased in GBM patients, it would be reasonable to expect to be able to isolate sufficient cells to undertake adoptive immunotherapy.

Primary, circulating DC are attractive candidates for next-generation adoptive immunotherapy. They are functionally superior to moDC^42^ and do not require lengthy *ex-vivo* differentiation, with vital cost and time implications. Clinical development of cDC2 for cancer immunotherapy is already in phase I trials and is resulting in some exciting clinical responses.^22,30^ Future studies may also combine this approach with immune checkpoint blockade. The importance of DC function for checkpoint blockade was established with mice deficient in cDC that failed to respond to checkpoint inhibitors.^43^ In humans, histological analysis of PD-L1 treated melanoma patients showed responders had pre-existing immunity against the tumor. This was defined as a tumor rich in CD8 + T-cells, an IFNγ CTL signature and PD-L1 expression.^44^ Hence using DC vaccination to increase the pool of antigen-specific CTL is likely to increase responses to anti-PD-1/PDL-1 antibodies. Our current investigations therefore highlight the potential of combining p38i-treated cDC2 with clinical immune checkpoint blockade antibodies. This is of particular importance given that PD-1 inhibitor monotherapy has failed to show a survival benefit in GBM patients.^45^

In summary, we describe suppression of the number, phenotype, and function of circulating DC subsets in patients with GBM. Furthermore, patients’ cDC2 cells had an immature phenotype and only weakly stimulated lymphocyte proliferation. Dexamethasone and tumor are shown to be causative factors contributing to DC dysfunction and importantly, the loss of function was recovered by inhibiting p38 MAPK prior to DC activation. We therefore propose the next generation of DC vaccines in GBM include enhanced p38i cDC2. In this regard, we will now undertake a clinical trial of p38 MAPK-inhibited cDC2 cells in adults with advanced cancer, the results of which will provide key insights into the immune system of these patients.

## Patients and methods

### Healthy donors and GBM patients

Studies were undertaken with ethical approvals granted by The University of Nottingham Research Ethics Committee; reference: 10/H0405/6, BT20052010, and 09/H0408/75. This study’s involvement with human subjects complies with the Declaration of Helsinki.

Adult participants were recruited from Nottingham University Hospital National Health Service (NHS) Trust and selected based on a diagnosis of GBM. Healthy controls were selected to be age- and sex-matched to recruited patients. Participants were consented prior to sample collection and sample use was restricted to this study. Patient characteristics are shown in Table 1.

### Enumeration of circulating dendritic cell subsets

To measure the abundance of circulating subsets of DC, freshly derived heparinized whole blood was incubated with combinations of appropriate test or control antibodies. The test antibodies included CD14-PerCP/Cy5.5 (325622), CD19-PerCP/Cy5.5 (302230), CD1c-APC (331524), CD141-APC (344106), CD303-FITC (354208 – all Biolegend), Slan (M-DC8)-FITC (130-093-178), HLA-DR-Vioblue (130-095-293 – both Miltenyi, UK), CD86-PE (12–0869-42) and PD-L1-PECy7 (25-5983-42 – both eBioscience). Respective isotype controls included IgG2a-FITC (400210), IgG1κ-APC (400122 – both Biolegend), IgG2a-Vioblue (130-094-671), IgM-FITC (130-093-178 – both Miltenyi), Ig2b-PE (12-4732-42) and IgG1κ-PECy7 (25-4714-42 – both eBioscience). Red blood cells were lysed in Red Cell Lysis buffer (eBioscience) and cells washed three times before re-suspension in MACSQuant Running Buffer (Miltenyi) and acquisition using a MACSQuant flow cytometer (Miltenyi).

The data was analyzed using FlowJo as follows. Briefly, following exclusion of doublet events, thresholds and box gates were objectively established by extensive use of isotype and fluorescence-minus-one (FMO) controls. Lineage negative (Lin-) (low side scatter, CD14-, CD19-), HLA-DR+ cells were selected for further analysis as follows: cDC2 cells were characterized as CD1c+, cDC1 subset was CD141+, and pDC were defined as CD303+ . The slanDC subset was 6-Sulfo LacNAc (Slan)+. A representative example of flow-cytometry gating is shown in Figure 1.

The absolute number of circulating DC per mL blood, and their relative frequency were calculated. Events were normalized by subtracting the number of events in FMO gates from test gates. Because of the scarcity of circulating DC subsets, consideration was given to the minimum number of acquired events for statistical validity.^46,47^ In consideration of variability of measuring rare cell events in whole blood we undertook a repeated analysis of control samples to ensure the assay delivered a coefficient of variance (CV) of approximately 10%.

### Isolation and culture of cDC2 cells

Peripheral blood mononuclear cells (PBMC) were separated from the whole blood using density centrifugation over Histopaque 1077 (Sigma, UK). Myeloid cDC2 cells were isolated using an anti-CD1c kit (Miltenyi) following the manufacturer’s instructions. Briefly, following depletion of B cells, CD1c+ cells were positively selected, washed, and counted. Cells were seeded in 96-well plates at 10^4^/well in RPMI-1640 with L-glutamine (Sigma, UK) containing 10% fetal calf serum (FCS), 1% sodium pyruvate (Sigma, UK) and 10 U/mL granulocyte-macrophage colony stimulating factor (GM-CSF, Peprotech, UK). Viability and purity were routinely determined to be >95%. Antibiotics were not used.

### Tumour-derived lysate (TL)

A panel of established human GBM cell lines (U87, LN18, LN229 obtained directly and recently from ATCC) were grown in 10-layer factory flasks (Corning Costar, Netherlands) and harvested by trypsinization after three days. The cells were then washed by centrifugation (300 g) and re-suspended at 10^7^/mL in RPMI-1640. Lysate was prepared from passage 5–8 by five cycles of freeze-thaw as previously described.^48^ All cells were routinely tested for mycoplasma prior to lysate preparation following the manufacturer’s protocol (R&D Systems 322361) and found to be negative.

### Response of cDC2 to Dex and TL

Immature cDC2 from healthy donors were plated in 96 well plates as described above. After resting for 30 min, the p38 MAPK selective antagonist BIRB0796 (Selleckchem, UK) was added to appropriate wells (1 μM) prior to addition of Dex (10^−6^M) or TL (ratio DC:TL 1:50). Cells were then incubated for 1 h prior to maturation with R848 2.5 μg/mL (InvivoGen, USA) plus poly I:C 20 μg/mL (Sigma, UK). Supernatants were harvested after 24 h and their cytokine content assayed. All experimental conditions were undertaken in triplicate.

### Cytokine ELISA

Supernatants were assayed by ELISA for IL-12 (BD Bioscience, UK) and IL-10 (R&D Systems, UK) according to the manufacturer’s instructions. Assays did not significantly cross-react with other proteins, and the sensitivities were 7.8 and 15 pg/mL, respectively.

### Western blotting

The activity of BIRB0796 and VX745 (Tocris) was confirmed by western blotting for the downstream target phospho-MK2. Isolated cDC2 cells were plated in DC medium containing GM-CSF. After resting, cells were treated with 1 μM BIRB0796 (or the indicated concentration of VX745) for 1 hr, stimulated with Poly I:C/R848 then lysed in radio-immunoprecipitation assay buffer (RIPA) (Sigma) containing protease inhibitor cocktail, phosphatase inhibitor cocktail 2 and 3 and Benzonase endonuclease (all Sigma) on ice for 1 h. Proteins were resolved by SDS-PAGE electrophoresis in 10% Tris/glycine gels and transferred to nitrocellulose. Membranes were blocked (5% milk powder (w/v) in PBST (PBS-0.1% (v/v) Tween 20)), probed with primary antibodies overnight at 4°C whose binding was detected with secondary antibodies in 1% milk powder (w/v) in PBST. Rabbit anti-human phospho-MK2(T334) and mouse anti-human β-actin (Sigma) were detected with donkey anti-rabbit-800 and anti-mouse-680 (LiCor) and imaged by LiCor Odyssey SA. Semi-quantitative analysis was undertaken with Odyssey software normalizing to β-actin.

### Mixed lymphocyte reaction (MLR)

The impact of inhibiting p38 MAPK on the ability of DC to drive T-cell proliferation was determined. DC were cultured with peripheral blood lymphocytes (PBL) in 96-well plates in T-cell medium (RPMI-1640, 10% FCS, non-essential amino acids, 4-(2-hydroxyethyl)-1-piperazineethanesulfonic acid (HEPES) and sodium pyruvate). Where specified, DC were co-cultured with Dex (10^−6^M) and TL (DC:TL 1:50). At day 5, tritiated thymidine (0.5μCi/well) was added during the final 18 h of culture and its incorporation determined using liquid scintillation counting. Supernatants were analyzed by ELISA for IFNγ (BD bioscience) and IL-10.

### Statistical analysis

Results were statistically analyzed using student’s t-test when normally distributed or normal when transformed using either ln x or 1/x. Otherwise, Mann–Whitney analysis was used for non-parametric data. Freidman tests were used, where multiple paired comparisons of non-parametric cytokine data were required and Wilcoxon test for single group paired analysis. Cut-off of P < 0.05 was interpreted as statistically significant.
